# ALS-ENABLE: creating synergy and opportunity at the Advanced Light Source synchrotron structural biology beamlines

**DOI:** 10.1107/S1600577525004205

**Published:** 2025-06-18

**Authors:** Corie Y. Ralston, Sayan Gupta, Joshua T. Del Mundo, Aimee Chi Soe, Brandon Russell, Behzad Rad, James Tyler, Sathi Paul, Darren N. Kahan, Line G. Kristensen, Simruthi Subramanian, Savannah Kidd, Kathryn Burnett, Banumathi Sankaran, Scott Classen, Daniil M. Prigozhin, John R. Taylor, Jeff M. Dickert, Kevin B. Royal, Anthony Rozales, Stacey L. Ortega, Marc Allaire, Jay C. Nix, Greg L. Hura, James M. Holton, Michal Hammel, P. D. Adams

**Affiliations:** a Molecular Foundry Division Lawrence Berkeley National Laboratory Berkeley CA94720 USA; b Molecular Biophysics and Integrated Bioimaging Division Lawrence Berkeley National Laboratory Berkeley CA94720 USA; c Department of Molecular and Cell Biology, Department of Chemistry, California Institute for Quantitative Biosciences University of California Berkeley Berkeley CA94720 USA; d Molecular Biology Consortium Lawrence Berkeley National Laboratory Berkeley CA94720 USA; e Department of Biochemistry and Biophysics University of California San Francisco CA94143 USA; f Structure Molecular Biology Group, Stanford Synchrotron Radiation Laboratory SLAC CA94025 USA; g Department of Bioengineering University of California Berkeley CA94720 USA; University of Manchester, United Kingdom

**Keywords:** macromolecular crystallography, small-angle X-ray scattering, X-ray footprinting mass spectrometry, synchrotron beamlines, mature synchrotron resource, structural biology, mature structural biology resource

## Abstract

ALS-ENABLE is an integrated NIH P30 resource at the Advanced Light Source synchrotron at Lawrence Berkeley National Laboratory in Berkeley, California, USA. The resource provides a single portal to the combined mature structural biology technologies of macromolecular crystallography, small-angle X-ray scattering and X-ray footprinting mass spectrometry, and includes beamlines 2.0.1, 3.3.1, 4.2.2, 5.0.1, 5.0.2, 5.0.3, 8.2.1, 8.2.2, 8.3.1 and 12.3.1. This paper describes the organizational structure and the technologies of ALS-ENABLE. A case study showcasing the main technologies of the resource applied to the characterization of the SpyCatcher–SpyTag protein system is presented.

## Abstract 2

A description of the beamlines within the structural biology ALS-ENABLE P30 resource at the Advanced Light Source synchrotron at Lawrence Berkeley National Laboratory is given, highlighted through the biophysical characterization of the SpyCatcher–SpyTag protein system.

## 1. Introduction

The Advanced Light Source (ALS) at Lawrence Berkeley National Laboratory (LBNL) is the world’s brightest soft X-ray synchrotron radiation source as well as a powerful source of hard X-rays. Each year it provides beam time to thousands of users covering a wide range of scientific projects. It is supported by the Department of Energy (DOE) Office of Basic Energy Sciences (BES), and as such is part of the national portfolio of user facilities. For structural biology work, the ALS hosts eight state-of-the-art macromolecular crystallography (MX) beamlines, one small-angle X-ray scattering (SAXS) beamline and an X-ray footprinting mass spectrometry (XFMS) beamline. The first ALS MX beamline was developed in 1996 from a wiggler insertion device source (Earnest *et al.*, 1996 ▸). Two fixed-energy side stations were built out from the same source in 2000. Not long after, the ALS developed the novel superbend magnet system to provide a compact, tunable and bright source of hard X-rays, which then allowed the build-out of additional MX beamlines starting in 2001 (MacDowell *et al.*, 2004 ▸). The SAXS station came online in 2004 as part of a first-of-its-kind combined MX/SAXS beamline (Classen *et al.*, 2013 ▸). The XFMS program, initially tested on beamline 5.3.1, moved to sector 3 in 2020 (Gupta *et al.*, 2020 ▸). The ten beamlines in ALS-ENABLE (ALS Efficient, Networked, Advanced Beamline Experiments) are managed by four groups: the Berkeley Center for Structural Biology (BCSB, beamlines 2.0.1, 5.0.1, 5.0.2, 5.0.3, 8.2.1 and 8.2.2), the Structurally Integrated Biology for Life Sciences (SIBYLS SAXS beamline 12.3.1), the Molecular Biology Consortium (MBC, beamline 4.2.2), the University of California’s MX beamline (8.3.1) and the XFMS program beamline (Approved Program with the Molecular Foundry, beamline 3.3.1). These structural biology groups have continuously upgraded their beamlines through the years, and the ALS has played key roles in helping bring the beamlines to their current level of maturity and providing efficient, up-to-date resources for the structural biology community (Table 1 ▸). Technology development at the beamline endstations has also kept pace with current trends, providing advanced tools for ever more challenging structural biology problems. For instance, the SAXS endstation on beamline 12.3.1 includes a high-throughput screening system for SAXS samples using a liquid handling automounter, as well as size-exclusion chromatography coupled with SAXS (SEC-SAXS) (Hura *et al.*, 2009 ▸; Hura *et al.*, 2013 ▸; Dyer *et al.*, 2014 ▸) or SEC with multi-angle light scattering coupled with SAXS (SEC-SAXS-MALS) (Rosenberg *et al.*, 2022 ▸). User samples for SAXS are run entirely by beamline staff, making for highly efficient operation. In the last few years, Pilatus detectors have been installed on most of the MX beamlines.

At the BCSB beamlines, high-throughput automated operation has enabled over 80% remote usage of the beamlines with very high efficiency (Zwart *et al.*, 2015 ▸), including automated fast raster- and vector-based data collection and screening, as well as the integration of automated crystal scoring, indexing and strategy calculation via *WebICE* (González *et al.*, 2008 ▸). The MBC beamline 4.2.2 allows shipping and remote data collection for room-temperature crystals. At beamline 8.3.1, innovative methods for simulating diffraction data (Holton *et al.*, 2014 ▸) and assessing radiation damage (Holton, 2009 ▸; Holton & Frankel, 2010 ▸; Zeldin *et al.*, 2013 ▸) are available. XFMS at beamline 3.3.1 has integrated inline fluorescence measurements with X-ray irradiation (Rosi *et al.*, 2022 ▸; Gupta *et al.*, 2025 ▸). All improvements to the storage ring, beamlines and endstations have had a positive cumulative impact on data quality. These ALS structural biology beamlines are state-of-the-art ‘mature’ resources, collectively serving hundreds of users annually and staffed by dedicated beamline scientists with strong long-term collaborations with NIH-funded PIs across the country. The MX, SAXS and XFMS scientists have worked together informally for years, but ALS-ENABLE brings them together into a formalized shared resource, taking advantage of the natural synergies between these groups and providing integrated structural biology solutions for the national biomedical research community.

## 2. Core technologies and beamline access

The goal of the ALS-ENABLE resource is to serve NIH funded investigators who run at the ALS under a general user (GU) proposal. All GU proposals for beam time at the ALS are peer-reviewed, scored and then sent to individual beamlines for allocation, with both a rapid (one month) track and a six-month track available. The system combines the MX, SAXS and XFMS beamlines into one ‘meta-beamline’ such that structural biology users need only apply through a single portal to access MX, SAXS and XFMS resources at the ALS. When users apply, they request ALS-ENABLE rather than a specific beamline, and can propose one or several of the structural biology methods. Users may also apply through the Molecular Foundry User Portal, and in this case can specify a structural biology beamline as part of their instrument request. Access through the Molecular Foundry system is most useful if projects involve protein engineering, crystallization, and/or access to LCMS data acquisition and analysis as part of an XFMS project. After proposal submission, projects are routed to the appropriate beamline and with the appropriate level of staff support, leaving no uncertainty on the part of the user as to which resource is best for their project. In addition, users may be introduced to new methods through this process, which often benefits their research programs.

User projects supported by ALS-ENABLE can be divided broadly into the four technology operations cores (TOCs) of the resource. TOC1, Rapid Response Crystallography, is designed to serve projects that can be handled in a high-throughput fashion. TOC2, High-Quality and High-Throughput SAXS, serves user projects requiring solution state information on conformation, and ties this information to the other two cores. TOC3, Specialized Crystallography, tackles crystallographic challenges reflecting the biology of large and flexible complexes. TOC4 provides access to the method of XFMS, which provides residue-level solvent accessibility information on proteins or protein complexes in solution. In addition, the Collaborative Crystallography program serves users by collecting diffraction data on behalf of users. Capabilities, modes of operation and sample requirements of each TOC are shown in Table 1 ▸. Further technical information on each TOC and beamline can be found on the resource website https://als-enable.lbl.gov.

## 3. Choice of the SpyCatcher–SpyTag system

Because of its versatile use in protein engineering applications, we choose the SpyCatcher–SpyTag protein to showcase the technologies in ALS-ENABLE. The SpyCatcher system derives from the collagen adhesion domain of *Streptococcus pyogenes*, in which an internal irreversible isopeptide bond spontaneously forms between the side chains of a lysine and an aspartate residue (Kang & Baker, 2011 ▸). In 2012, the Howarth group split the protein into the so-called SpyCatcher001 domain, containing the lysine residue, and the SpyTag001 peptide, containing the aspartate (Zakeri *et al.*, 2012 ▸). They showed that, when the two are mixed in solution, the covalent isopeptide bond spontaneously forms on a timescale of minutes at micromolar concentration, recapitulating the original protein. The group later used a phage display platform and selective mutation targeted towards enhancing electrostatic complementarity to develop the SpyCatcher002 and SpyCatcher003 variants, each with higher affinity and speed of isopeptide bond formation (Keeble *et al.*, 2019 ▸). SpyTag003 included mutations to add positive charge to the N-terminus in order to stabilize the interaction with a patch of negative charge on SpyCatcher. Further mutations included loop stabilization and strengthening of additional electrostatic interactions, and led to a second-order rate constant of 5.5 × 10^5^*M*^−1^ s^−1^, which was 400-fold faster than the original SpyCatcher001–Tag001 system. Since that time, the SpyCatcher-Tag system has proved useful for biological imaging using fluoro­phores attached to either SpyCatcher or SpyTag, and either endogenously expressing the proteins or delivering through the cell membrane (Keeble & Howarth, 2020 ▸; Charrier *et al.*, 2019 ▸; Hatlem *et al.*, 2019 ▸; Tyler *et al.*, 2023 ▸).

Below we describe the characterization of the SpyCatcher003–SpyTag003 protein system using the main technologies of the ALS-ENABLE resource. We first crystallized and solved the structure of the SpyCatcher003–SpyTag003, which had not previously been solved. We collected SAXS on the same system to understand the solution state flexibility of the system. We then further characterized the binding of SpyTag003 to SpyCatcher003 using FRET inline with XFMS in a newly designed mixing cell at the footprinting beamline endstation. Together, these data provide a comprehensive picture of the SpyCatcher003–Tag003 system from the atomic level crystal structure to its flexibility in solution and the residue-level interactions driving the formation of the SpyCatcher–SpyTag complex.

## 4. Materials and methods

### 4.1. Protein purification and labeling

SpyCatcher003 and SpyCatcher003-A49C were over-expressed in BL21(DE3) pLysS strains according to previously published protocols (Zakeri *et al.*, 2012 ▸; Keeble *et al.*, 2019 ▸; Tyler *et al.*, 2023 ▸). Briefly, cells were grown to mid-log phase (0.6–0.8 OD 600 nm) and then induced with 0.5 m*M* IPTG. After 3 h of induction, cells were harvested by centrifugation at 8000 rpm for 20 min at 4°C in a JLA 8.1 rotor.

Cell pellets were lysed using C3 Emulsiflex homogenizer (Avestin Inc. Otowa, ON, Canada) at 18000–20000 psi. The lysate was clarified using a Beckmann Optima X-100 Ultra centrifuge at 100000*g* for 60 min. The clarified lysate was run over an FF NiNTA column (Cytiva) on an AKTA Pure system (Cytiva). The N-terminal 6xHis tag was removed by incubating overnight at 4°C with 100-fold excess of TEV protease. The cleaved tag was removed by adding Ni-NTA Agarose (Qiagen) for 30 min and separated with centrifugation at 4000 rpm at 4°C for 15 min. Cleaved proteins were quantified using UV–Vis spectroscopy on a Perkin Elmer Lambda 360 using the extinction coefficients calculated for each sequence by *ExPasy ProtParam*.

Before labeling, the SpyCatcher003-A49C protein was dialyzed to remove any reducing agent (TCEP or DTT). Alexa 555 male­imide dye was added to a final concentration 2× that of the protein concentration and equilibrated at 4°C for 4 h. The reaction was then run over a BioGel P-6 column equilibrated in 1X PBS. The labeling efficiency was determined by UV–Vis using the dye concentration calculated at maximum absorbance (AF555, ɛ556 nm = 158000 *M*^−1^ cm^−1^), taking into account the dye’s correction factor (CF280 = 0.08 for AF555). The degree of labeling was determined by dividing the dye concentration by the protein concentration. SpyTag003 and SpyTag003-sfGFP were purchased from AnaSpec Inc. in lyophilized form. Peptides were resuspended in 1X PBS.

### 4.2. Sample preparation and data collection

#### 4.2.1. MX

The SpyCatcher003–SpyTag003 complex was formed by incubation at 1:1 stoichiometry overnight and then concentrated to 50 mg ml^−1^ in Tris buffer (pH 8.0) with 50 m*M* NaCl. Initial sitting-drop screening trays were prepared using a Phoenix crystallization robot (Art Robbins Instruments) and standard screens. Crystals grew to 20 µm plates in 30.00% *w*/*v* polyethyl­ene glycol 8000; 200 m*M* ammonium sulfate; 1.00 m*M* Anderson–Evans polyoxotungstate ‘TEW’ additive (Blazevic & Rompel, 2016 ▸) after 2 months. Further optimization led to 50 µm rectangular crystals, with the best diffracting crystals found in the original crystallization conditions in a hanging drop with 2.0:1.5 µl protein:reservoir buffer ratio. Crystals were transferred to a 20% glycerol solution before flash-freezing. Diffraction data were collected at 1 Å using the automated pipeline at ALS beamline 2.0.1, which included fully automated raster screening, data collection and *XDS* processing. The presence of TEW in the molecular structure enabled the use of molecular replacement combined with SAD (MRSAD) phasing off the anomalous signal from the W atoms using the *Phenix* program (Liebschner *et al.*, 2019 ▸). The isopeptide bond between the side-chain nitro­gen of Lys31 on SpyCatcher and the gamma carbon of Asp117 on SpyTag was clearly visible in the density and added as a geometry constraint in the final refinement. The crystal structure has been deposited to the PDB (PDB entry 9oj3) and the raw diffraction files have been deposited at https://proteindiffraction.org/.

#### 4.2.2. SAXS

We collected experimental SAXS data from a monodisperse state, free of higher oligomeric states and aggregation. We applied SEC-SAXS. Data were collected on SIBYLS beamline 12.3.1 at the ALS with the recently developed SEC-SAXS-MALS modality (Rosenberg *et al.*, 2022 ▸). The X-ray wavelength was set at λ = 1.127 Å and the sample-to-detector distance was 2100 mm, resulting in scattering vector magnitudes, *q*, ranging from 0.01 Å^−1^ to 0.47 Å^−1^. 60 µl of sample with a concentration of ∼3 mg ml^−1^ was prepared in the SEC running buffer. The Shodex KW803 column was equilibrated with a running buffer with a flow rate of 0.65 ml min^−1^. Each sample was injected in an SEC column, and two-second X-ray exposures were recorded continuously for 24 min. BioXTAS RAW (Hopkins *et al.*, 2017 ▸) was used for further SEC-SAXS processing, including buffer subtractions and merging SAXS frames across the elution peak. The final merged SAXS curves were further used for Guinier analysis using RAW (Hopkins *et al.*, 2017 ▸) and computing *P*(*r*) functions by the program *GNOM* (Svergun, 1992 ▸). The MW_SAXS_ was calculated by volume of correlation (Rambo & Tainer, 2013 ▸) and compared with the molecular weight estimated by SEC-MALS. The SEC-SAXS data were deposited in the SIMPLE SCATTERING database https://simplescattering.com. Atomistic models were built using *AlphaFold2* (Bryant *et al.*, 2022 ▸). Conformational sampling for SAXS fit optimization was performed by *BILBOMD* (Pelikan *et al.*, 2009 ▸). SAXS fitting and ensemble model selections were done using *FoXS* (Schneidman-Duhovny *et al.*, 2013 ▸) and *Multi-FoXS* (Schneidman-Duhovny *et al.*, 2016 ▸).

#### 4.2.3. XFMS

The XFMS sample delivery method using a jet or capillary has been described previously (Rosi *et al.*, 2022 ▸). For the current study, we used the inline mixing cell to enable the kinetics evaluation of protein systems by both spectroscopy and XFMS simultaneously. The SpyTag003-sfGPF and SpyCatcher003-AF555 samples, each at 10 µ*M* concentration, were mixed with delays set to 0.02, 0.05, 0.1, 0.4, 1, 5 and 10 s before probing by fluorescence emission spectroscopy and X-ray exposure. The sample flow speed through the X-ray beam was set to a fixed value for homogenous mixing for all the mixing delays and to maintain a constant X-ray exposure of 250 µs. The fluorescence from the mixed samples was collected inline from the capillary immediately above the X-ray impingement point, approximately 100 µs prior to X-ray exposure. Exposed samples were rapidly quenched in the fraction collector and processed by LCMS, as previously described (Rosi *et al.*, 2022 ▸). For residue-specific LCMS analysis, only the extracted ion chromatograms with a signal-to-noise ratio >1000 and a mass accuracy of 20 p.p.m. together with validated MS/MS sequence assignment were considered for quantitative analysis. The total number of hydroxyl modified residues reported was 14, which is approximately 12% of the full sequence of SpyCatcher003–SpyTag003. The details of time-resolved hybrid FRET-XFMS are described in the supporting information. Details on the XFMS method, which uses sites of hydroxyl radical modification to determine solvent accessibility changes, have been previously described (Gupta *et al.*, 2007 ▸).

## 5. Results and discussion

### 5.1. Crystallography

**Figure 1:**
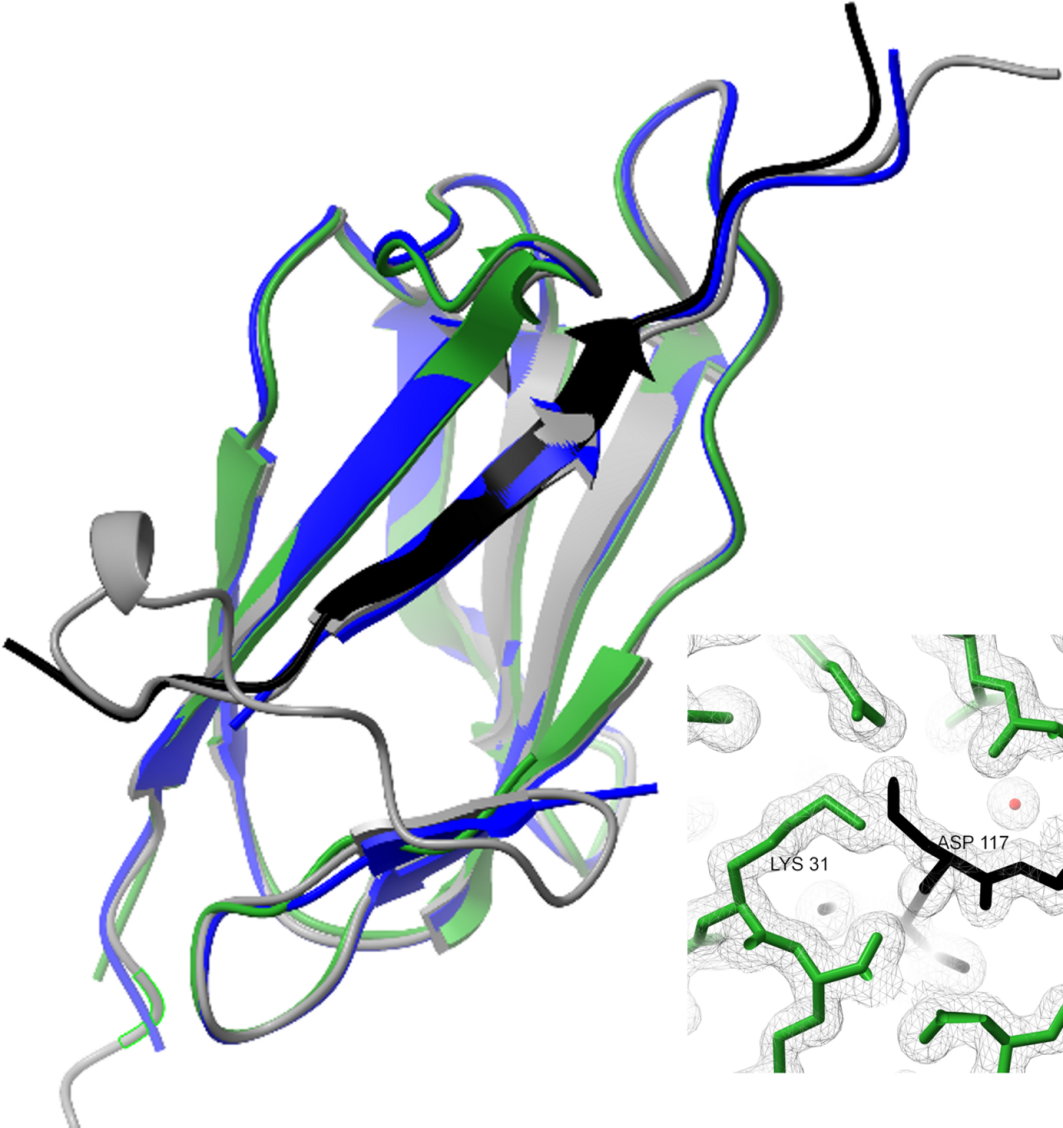
SpyCatcher-Tag structures. Dark blue: previously determined crystal structure of SpyCatcher001–SpyTag001 (PDB entry 4mli; Li *et al.*, 2014 ▸). Green: SpyCatcher003–SpyTag003. Gray: *AlphaFold* model of SpyCatcher003–SpyTag003. Inset: isopeptide bond in SpyCatcher003–SpyTag003 structure between Lys31 NZ atom in the Catcher domain (green) and Asp117 CG atom in the Tag peptide (black).

The SpyCatcher003–SpyTag003 crystal structure closely matches the two previously determined SpyCatcher001–SpyTag001 structures, 4MLS and 4MLI (Li *et al.*, 2014 ▸) (Figs. 1 ▸ and 2 ▸). In all structures, the isopeptide bond distance between the nitro­gen atom of Lys31 and the gamma carbon atom of Asp117 is within 1.4 Å. The 4MLS crystal structure was obtained from a construct missing 22 N-terminal residues, while the 4MLI structure was obtained from a construct containing these residues. However, those residues were not resolved in either the 4MLI structure or the current SpyCatcher003–SpyTag003 structure, and the *AlphaFold* models of both the −001 and the 003 constructs predict that these residues are unstructured. Compared with the SpyTag001 constructs, the SpyTag003 version contains three additional residues at the N-terminus which are visible in the density.

The SpyCatcher003–SpyTag003 system was previously engineered from SpyCatcher001–SpyTag001 with the aim of speeding the Catcher–Tag binding interaction to a rate approaching the diffusion limit (Keeble *et al.*, 2019 ▸). Towards that goal, positive charge was introduced to the N-terminus of SpyTag003 in the form of R108 and G109 to promote interactions with the negatively charged region defined by E20, E21, D22 and E96 in the SpyCatcher domain. To compensate for the additional positive charge in SpyTag003, the mutations K37R, Q62H, A89P, T91E and Q97D were introduced into SpyCatcher003. A89P was also engineered to reduce flexibility in the 79–89 loop which lines one side of the SpyTag docking region. These changes do not affect the overall fold, as shown by the similarity of the 001 and 003 structures, pointing to the stability of the Catcher–Tag complex.

### 5.2. SAXS

**Figure 3:**
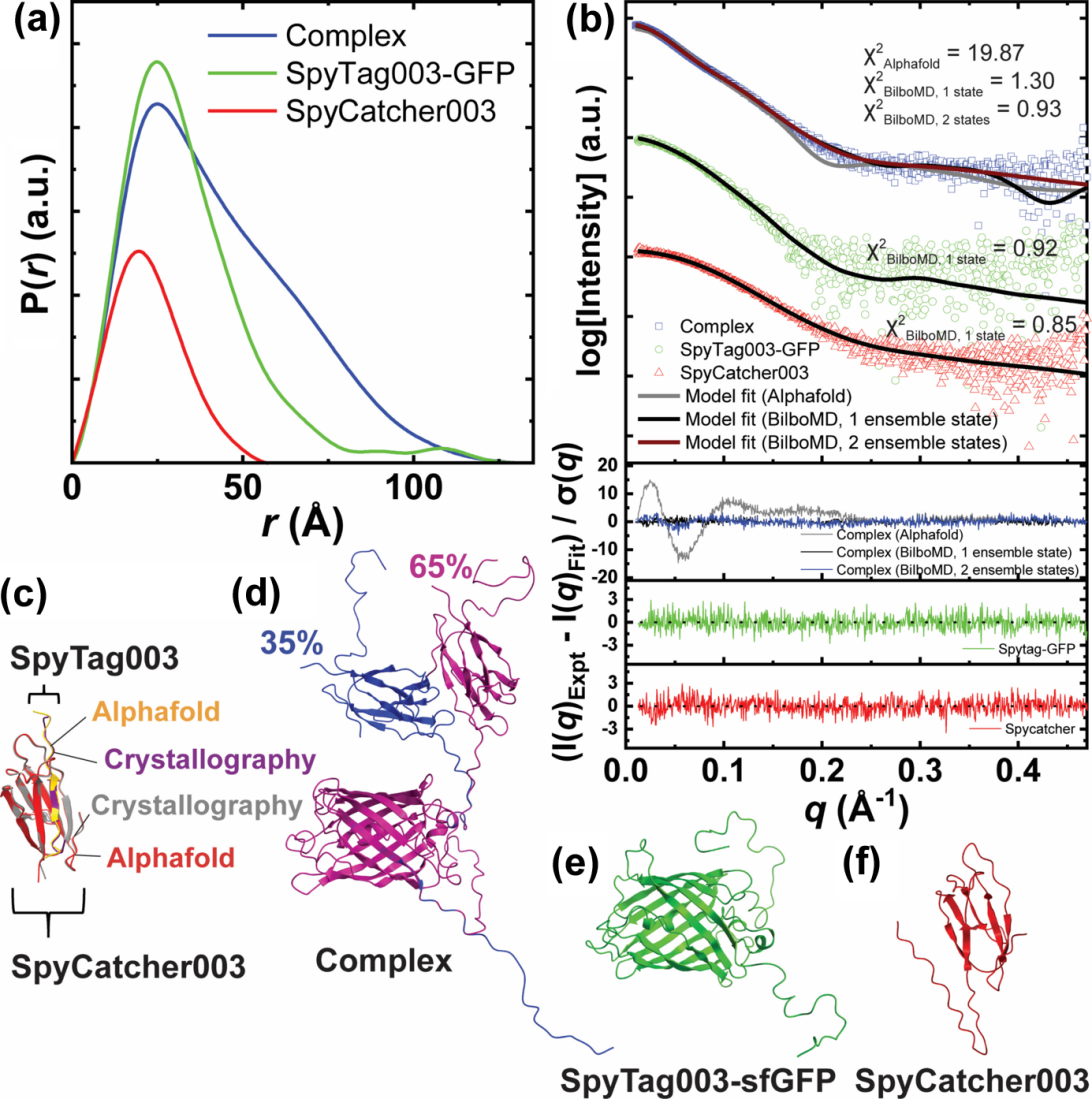
SAXS on SpyCatcher003-SpyTag003. (*a*) Pair distribution functions, area-normalized by molecular weight, and (*b*) SAXS curves (offset) and residuals of the SpyCatcher003–SpyTag003-sfGFP complex (blue), SpyTag003-sfGFP (green) and SpyCatcher003 (red). The gray curve indicates the fit by the *FoXS* of the *AlphaFold* prediction of the complex. Black curves indicate the fits by *FoXS* from *BilboMD*-fitted models. (*c*) Comparison of the crystallography complex (PDB entry 4mli) with the *AlphaFold * prediction. (*d*)–(*f*) Best fit conformations of (*d*) SpyCatcher003–SpyTag003-sfGFP complex, 2-state ensemble; (*e*) SpyTag003-sfGFP, 1-state ensemble; and (*f*) SpyCatcher003, 1-state ensemble, after *BilboMD* fitting.

We collected SAXS data in SEC-SAXS-MALS mode (Rosenberg *et al.*, 2022 ▸) of SpyTag003-sfGFP, SpyCatcher003 and its complex. The MW calculated from SAXS clearly shows the monomeric state of the proteins (Table 2 ▸). The *P*(*r*) of both the SpyTag003-sfGFP and the SpyTag003-sfGFP–SpyCatcher003 complex show a long tail, which indicates the presence of an unfolded portion of the proteins [Fig. 3 ▸(*a*), Table 2 ▸]. The C-terminus was determined to be unfolded, and the SpyTag003 region in SpyTag-sfGFP was determined to be solvent-exposed, based on our interpretation from atomistic modeling [Figs. 3 ▸(*b*)–3(*e*)]. The presence of the unfolded C-terminal regions of SpyTag003-sfGFP and the unfolded N-terminal region of SpyCatcher003 in a complex state was also required to match the experimental SAXS data [Figs. 3 ▸(*b*) and 3 ▸(*d*)]. The atomistic modeling of the SAXS data revealed that the compact conformation of the *AlphaFold* model of the complex does not fit the experimental SAXS data, with a poor fit χ^2^ = 19.87. The single-state best-fit model of the complex derived by conformational flexing (see Methods[Sec sec4]) of the SpyCatcher003 position relative to the GFP significantly improved the fit, with χ^2^ = 1.30. However, further fit improvement was observed by selecting the 2-state model, χ^2^ = 0.93 [Fig. 3 ▸(*d*)], pointing to the flexible connections between SpyTag003-sfGFP and SpyCatcher003. The weighted conformations of the open and closed conformations, as shown in Fig. 3 ▸(*d*), do not represent the only conformations in solution and should be considered as an approximation of conformational space. The *AlphaFold* model of SpyCatcher003 shows a similar fold with the crystal structure of SpyTag001–SpyCatcher001 [Fig. 3 ▸(*c*)]. However, to match the SAXS data of SpyCatcher003, it was necessary to optimize the conformation of the N-terminal unfolded region to provide a more realistic plastic conformation of this region rather than stiffly extended conformation as derived by *AlphaFold* [Figs. 3 ▸(*c*) and 3 ▸(*f*)]. The data and models are available in the Simple Scattering database.

### 5.3. Time-resolved hybrid FRET-XFMS

In order to understand the global and local structural dynamics of the association of SpyCatcher003 with SpyTag003, we used millisecond mixing experiments, simultaneously measuring FRET between the SpyCatcher003-AF555 and SpyTag003-sfGFP while collecting XFMS data on the same mixed sample for determination of site-specific local solvent accessibility changes. The association kinetics of the split protein pair was previously studied using fluorescence and gel electrophoresis and shown to be dependent on their respective concentrations (Keeble *et al.*, 2019 ▸). The delay time range of 0.02 s to 10 s was suitable to monitor the complete association kinetics at ∼5–10 µ*M* concentration of the split protein pair. At each time delay, we collected 4–6 data points for both FRET and LCMS for statistical averaging. The fixed exposure of ∼250 µs generated a quantifiable yield of side-chain modification for XFMS analysis. This X-ray exposure was within XFMS’s optimal exposure time range, which was determined by our standard Alexa488 assay (Rosi *et al.*, 2022 ▸; Gupta *et al.*, 2007 ▸).

**Figure 4:**
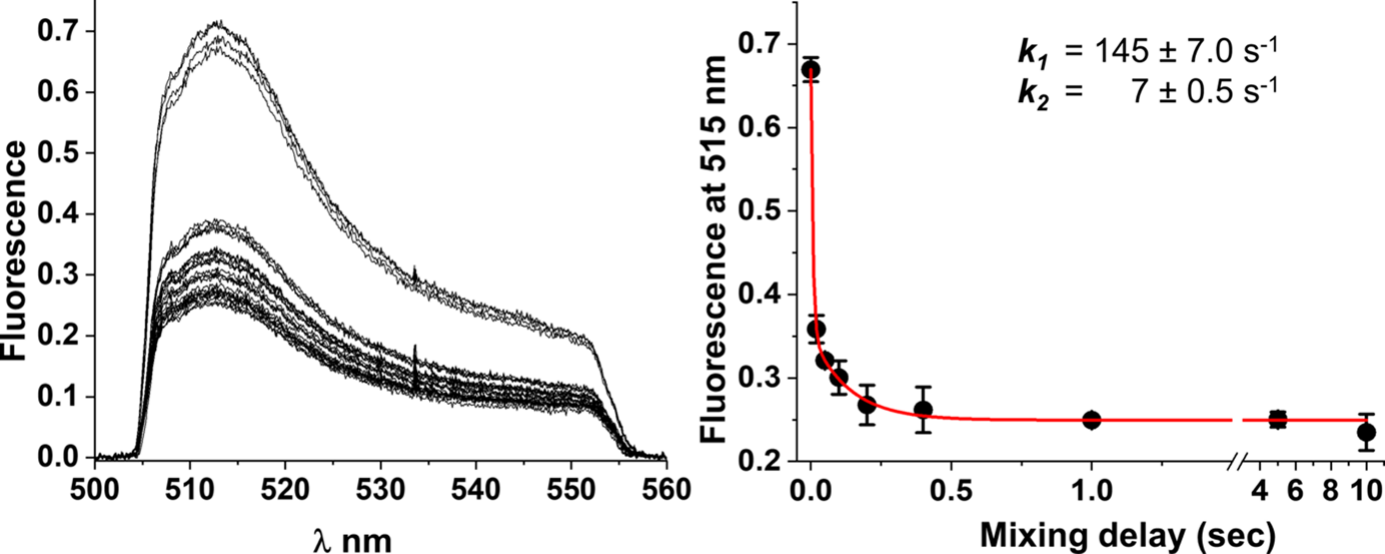
Time-resolved hybrid spectroscopy XFMS. The decrease in the fluorescence emission of SpyTag003-sfGFP during the formation of the SpyCatcher003-AF588–SpyTag003-sfGFP complex is due to FRET (left). Kinetic trace of fluorescence emission at 515 nm is best fitted to a double exponential decay (solid line) that determines a fast and slow rate of FRET response during the binding event (right).

The donor–acceptor pair SpyTag003-sfGFP and SpyCatcher003-AF555 show strong FRET, a distance-dependent, non-radiative energy transfer process from the excited state of a donor molecule to the ground state of an acceptor molecule by a dipole–dipole coupling interaction within 10 nm proximity (Lakowicz, 2006 ▸; Ma *et al.*, 2014 ▸) in response to complex formation and isopeptide bond formation. When mixing an equimolar amount of SpyTag003-sfGFP and SpyCatcher003-AF555, we observed >50% reduction of the fluorescence of SpyTag003-sfGFP over a period of 20 ms to 10 s (Fig. 4 ▸). The FRET response confirms the time evolution of the proximity of SpyTag003-sfGFP and SpyCatcher003-AF555. The FRET trace was best fitted to a double exponential decay with ∼80% of the amplitude of change due to a fast rate of 143 s^−1^, and a slow rate of conformation change of 7 s^−1^. In contrast, the stopped-flow kinetics study with a similar donor–acceptor system reported earlier was best fit with a triple exponential, with one fast rate of conformational change accounting for the majority of the amplitude of FRET change and two other slower rates of conformational change (Keeble *et al.*, 2019 ▸). Although we used ∼50 times higher protein concentration than in the previous study, interestingly, in both studies, the rate of the fast phase of conformational change is 20–40 fold higher than that of the slow phase, which indicated that the split-protein pair followed similar, if not identical, association kinetics. The difference in double versus triple exponential curve fitting is most likely due to the limited number of time delay data points for the time-resolved hybrid FRET-XFMS experiment compared with the continuous FRET data collected from the stopped-flow instrument, rather than the difference between the fluorescence tagging of SpyCatcher003 with mClover3 versus sfGFP, or differing concentrations of the components. Our FRET data could be further improved by increasing the number of mixing delays and concentration-dependent kinetics traces, which is beyond the scope of the current study.

**Figure 5:**
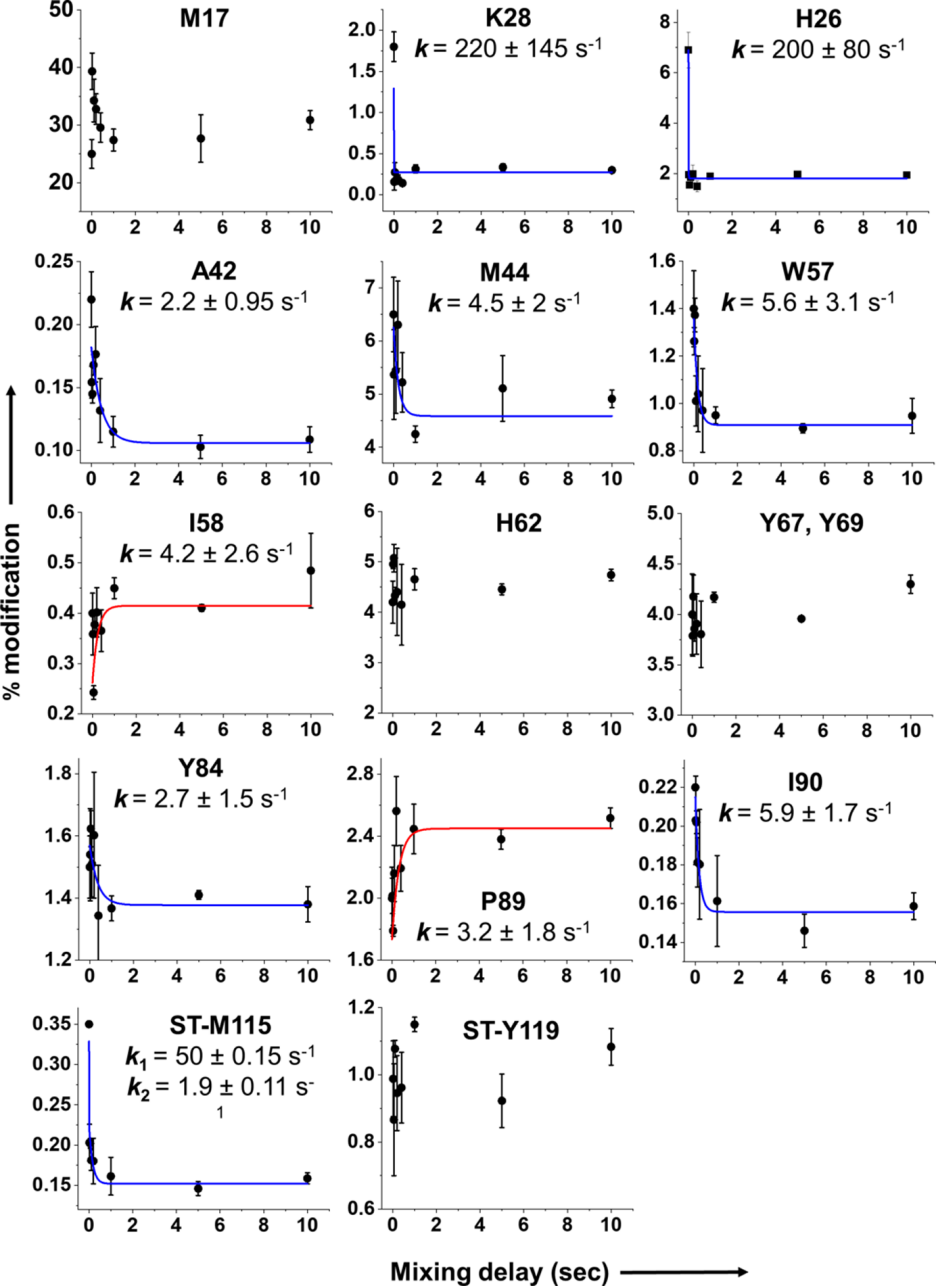
Time course of residue-specific solvent accessibility changes. The dots represent mean percentage modification ± the standard error from five or six independent measurements for each delay time point. The solid line represents a single or double exponential fit of the time course of the percentage modification, which provided site specific rate constants of conformation change. Blue and red indicate the decrease or increase in accessibility, respectively, upon SpyTag003 binding to SpyCatcher003.

**Figure 6:**
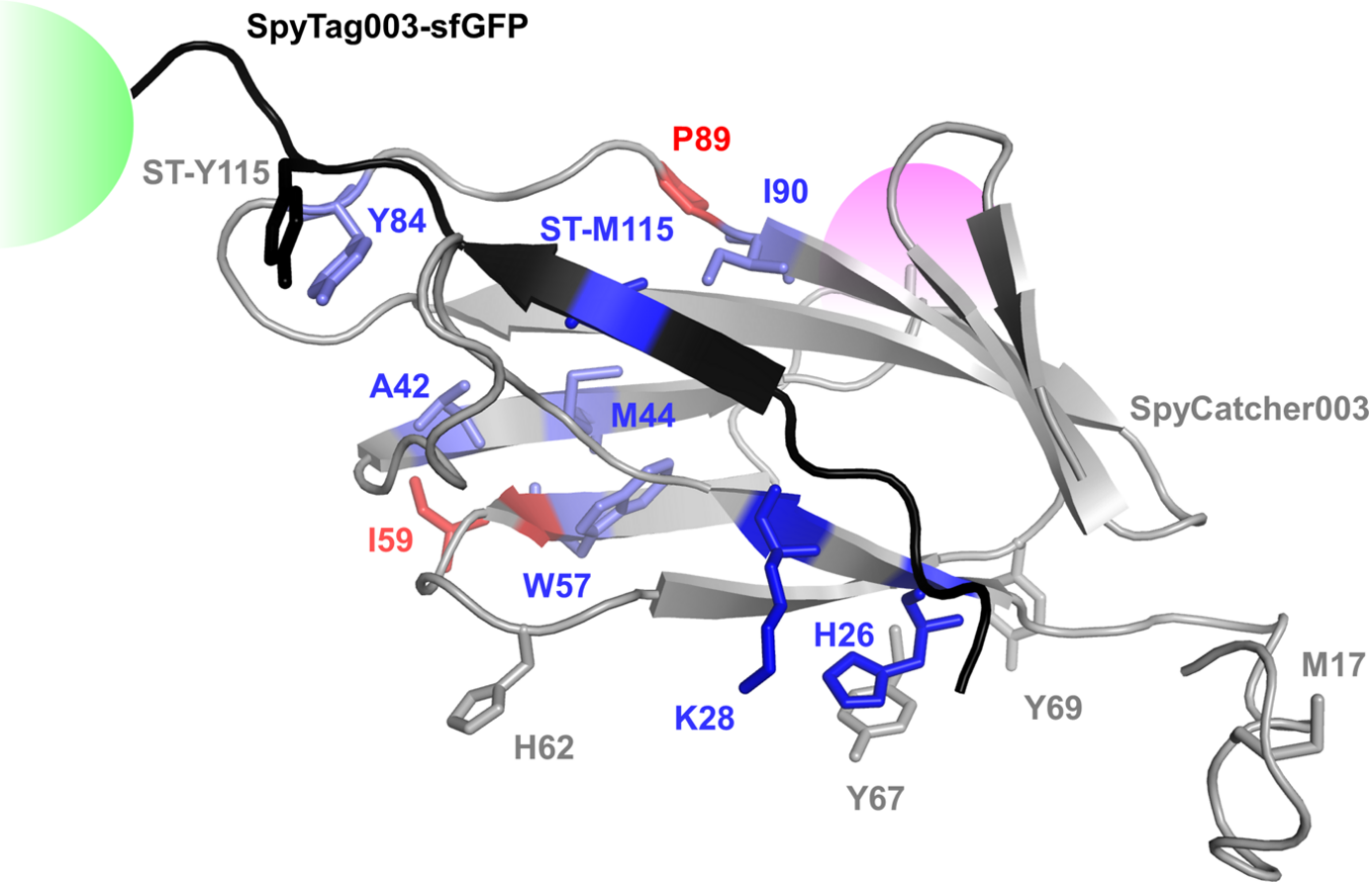
Residue-specific speed of solvent accessibility change. The crystal structure of SpyCatcher003 (gray) was extended to include additional C-terminus residues by homology modeling using PDB entry 2x5p (Oke *et al.*, 2010 ▸), the original fibronectin binding protein from which SpyCatcher was derived. Residues in light blue and red indicate a slow rate of decrease and increase, respectively; residues in deep blue indicate a fast rate of decrease; residues in gray did not show any change in solvent accessibility during binding of SpyTag003 to SpyCatcher003. SpyTag003 is shown in black. The location of the attached fluorescence molecules, sfGFP and Alexa 555, attached to the C-terminal of SpyTag003 and Cys49 of SpyCatcher003, respectively, are shown in green and pink.

**Figure 7:**
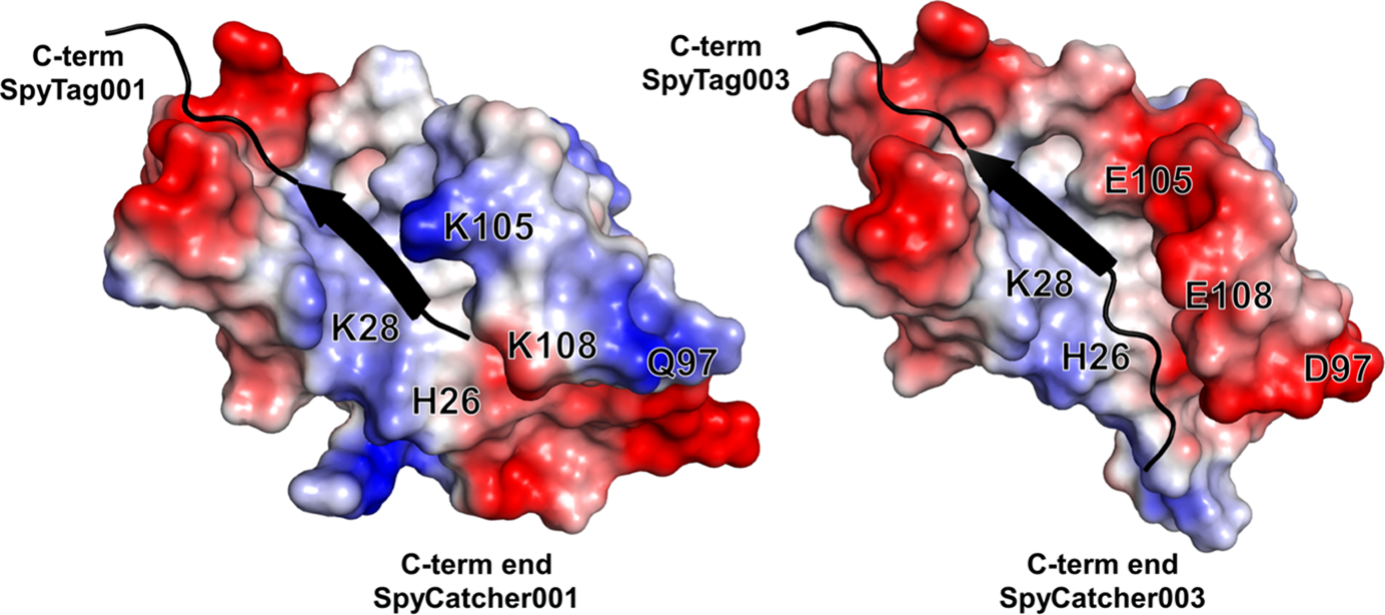
Differences in electrostatic potential maps. The SpyTag001 and SpyTag003 structures superimposed on the electrostatic potential map of the homology-modeled extended sequence of SpyCatcher001 (left) and SpyCatcher003 (right), respectively. The three mutations, Q97D, K105E and K108E, make a significant surface polarity switch towards the N-terminal section of SpyTag003.

The inline fluorescence collection was positioned immediately above the X-ray impingement point on the capillary, resulting in a less than 100 µs delay between the fluorescence collection and the X-ray irradiation. Thus, our XFMS setup captures the same event as the FRET response to within 100 µs and can be used to capture local kinetics information during conformational changes essentially simultaneously with the global information obtained by FRET. X-ray exposure times contributed negligibly to the overall mixing delays and were also well below the threshold to cause significant radiation damage-induced conformational perturbation. For LCMS analysis, experiments in replicate for all mixing delays exhibited consistent radiolytic labeling on identical residues. The site-specific kinetic traces, represented by % modification versus delay time and their positions on the structure are shown in Figs. 5 ▸ and 6 ▸, respectively. The kinetic traces of the solvent accessibility changes in SpyCatcher003 were fitted with a single exponential function. After mixing, we observed single exponential decay in the percentage of modification at SpyCatcher003 residues A42, M44, W57 and Y84. These residues are within 4–5 Å of the SpyTag003 binding site, and line the interior hydro­phobic core of SpyCatcher003 (Fig. 6 ▸). The protection of these residues is indicative of a closing of the core by SpyTag003. The rate of conformation changes of these residues ranges from 2 to 5 s^−1^, roughly equal to the slow rate of global conformation changes obtained from the FRET analysis. Two residues, K28 and H26, showed a rapid decrease in solvent accessibility in the burst phase of the kinetic traces with an estimated rate similar to the fast phase of the global measurements. These two residues are located in close proximity to the SpyTag003 N-terminal residues R108, G109 and V110, which were added to the SpyTag peptide when designing SpyTag003 from the 001 and 002 variants (Keeble *et al.*, 2019 ▸). The tighter affinity of the interaction reported earlier between SpyCatcher003 and SpyTag003, as well as the fast kinetic phase, detected both globally and locally in the current study, might be associated with the added stabilization of the activation state by the engineered electrostatic interactions. In addition, the introduction of negatively charged polar residues around this location of SpyCatcher003 (Q97D, K105E and K108E) and the positively charged residue on SpyTag003 (R108) (Fig. 7 ▸) may have led to an increase in propensity to form an hydrogen-bonding network. As an internal control, time-resolved measurement showed no changes in the SpyCatcher003 residues M17, H62, Y67 and Y69. These residues are not located at the binding interface. In contrast, SpyCatcher003 residues I58 and P89 showed an exponential increase in solvent accessibility with a rate of conformational change of 3–4 s^−1^. These reciprocal conformation changes relative to that of W57 and I90, which showed a decrease in accessibility at a similar rate, indicate that there might be a reorientation of the side chains during the binding event at these locations. The SpyTag003-sfGFP residue M115, which is directly at the binding interface, showed a double exponential decay with a relatively fast (50 s^−1^) and slow (2 s^−1^) rate of conformational change. However, the SpyTag003-sfGFP residue Y119, known to interact with the SpyCatcher003 residue Y84, did not show any kinetic trace. Although the functional relevance of this data is unclear, the lack of clear kinetics might be due to conformational heterogeneity associated with the orientation of the large sfGFP attached to the SpyTag003, as indicated by SAXS.

Overall, the hybrid time-resolved data suggested location-specific fast and slower phases of conformational dynamics. The fast conformational transition may be associated with charge–charge stabilization, which lowers overall activation energy and kinetically drives interactions faster. The slower phase of conformational rearrangement is likely to be mostly mediated by the stabilization of the complex by hydro­phobic interactions.

## 6. Conclusions

The goal of ALS-ENABLE is to provide an integrated, efficient synchrotron structural biology resource for the research community that optimizes the chances of successfully obtaining important structural and dynamics information on biomolecules and their complexes. To showcase the synergy of the methods available through ALS-ENABLE, we collected structural information on the SpyCatcher–SpyTag system using each method in the resource. We used MX to obtain the crystal structure of the SpyCatcher003–SpyTag003 complex, validating the *AlphaFold* predicted model, and showing that the mutations introduced to produce the 003 system from the 001 system did not substantially change the folded conformation of the protein. SAXS characterized the orientation of SpyCatcher to the sfGFP construct as attached to SpyTag. With the XFMS method, we gathered inline FRET data during X-ray irradiation and reported on both global structural changes and residue-specific structural changes in solution during the binding of SpyTag003 to SpyCatcher003, validating previous FRET measurements on the 003 system and providing a rationale for the greater stability engineered into the 003 system. Overall, these results demonstrated the utility of combining static (crystal) data with dynamic (SAXS and XFMS) data in order to obtain a complete biophysical picture of a protein system.

## Supplementary Material

Sections S1 to S3 including Figure S1. DOI: 10.1107/S1600577525004205/he5689sup1.pdf

Structure factors: contains datablock(s) spycatcher-MX_refine. DOI: 10.1107/S1600577525004205/he5689sup2.hkl

## Figures and Tables

**Figure 2:**
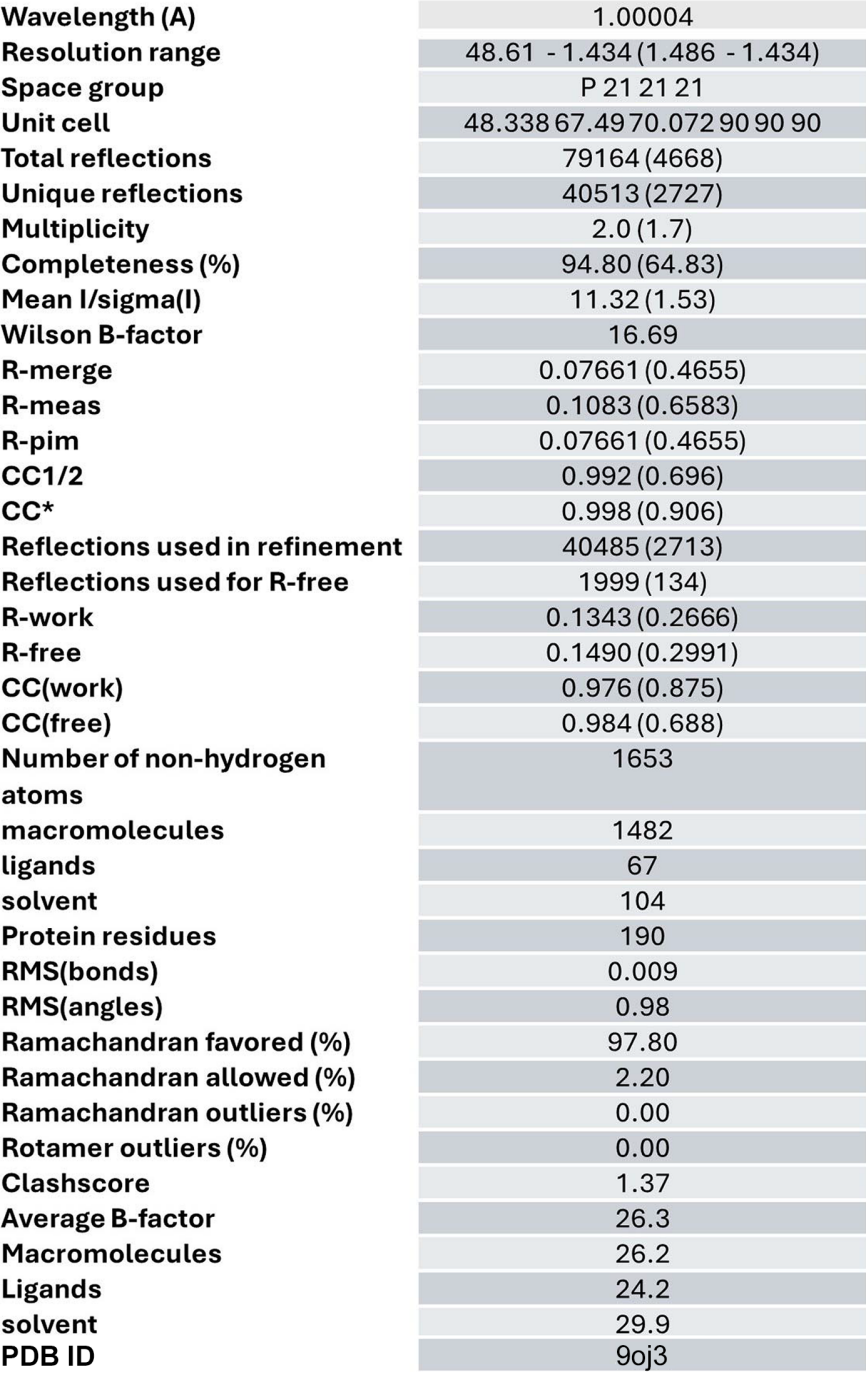
Data collection and refinement statistics.

**Table 1:** ALS-ENABLE beamlines and parameters Multilayer: ML; root-mean-square: RMS.

	2.0.1	3.3.1	4.2.2	5.0.1	5.0.2	5.0.3	8.2.1	8.2.2	8.3.1	12.3.1
Detector	Dectris Eiger2 16M	N/A	RDI CMOS 8M	Dectris Pilatus3 2M	Dectris Pilatus3 6M	Dectris Pilatus3 2M	Eiger2 9M	Dectris Pilatus3 2M	Dectris Pilatus 6M	Dectris Pilatus 2M
Frame rate (Hz)	280	N/A	25	25	50	25	40	25	100	250
Fluorescence detector	Hitachi Vortex EM	Hamamatsu PMT2100	Hitachi Vortex VX100	None	Hitachi Vortex EM	None	Oxford Cyberstar NaI:Tl	Oxford Cyberstar NaI:Tl	Hitachi Vortex EX100	None
Source	Undulator (IVID)	Bend (1.27 T)	Super-Bend (5 T)	56-pole 11.4 cm wiggler (1.9 T)			Superbend (5 T)			
Primary mirror RMS slope error (µrad)	None	Silicon; 0.7	Silicon; 0.2	Silicon; 1.0	Silicon; 1.0	Silicon; 1.0	Silicon; 0.2	Silicon; 0.2	Silicon; 0.2	Silicon; 0.8
Energy range (keV)	5–15	2–16	7.0–15	12.7	5–16	12.7	5–16 ML: 10–13	5–16	5–17	5–17
Monochromator	Cryo Si(111); 0.4% ML	None	Sagittal Si(111)	Si(220)	Cryo Si(111)	Si(220)	Cryo Si(111); 0.4% ML	Si(111)	Si(111)	1.3% ML
Flux (photons s^−1^) 100 µm diameter hole	2 × 10^12^ ML 1 × 10^13^	1 × 10^16^	1 × 10^12^	3 × 10^11^	1.5 × 10^12^	5 × 10^11^	6 × 10^11^ ML 2 × 10^12^	6 × 10^11^	1 × 10^12^	4 × 10^13^
Focus size (*v* × *h*) (µm)	15 × 15	80 × 400	85 × 55	350 × 150	350 × 150	350 × 150	100 × 50	75 × 40	80 × 60	87 × 48
Collimation/defocus (µm)	15–100	1000 × 1000	20–400	20–150	10–150	20–150	20–150	20–150	15–100	5000 × 500
Damage limit (30 MGy)	7 s ML: 0.1 s	N/A	3.3 min	50 min	7 min	33 min	8 min ML: 150 s	5 min	5 min	0.3–10 s
Robot	NATX-ray GRob	Harvard Apparatus PHD 22/2000 Syringe pump	Rigaku ACTOR	Berkeley Auto-Mounter	Berkeley Auto-Mounter	Berkeley Auto-Mounter	Rigaku ACTOR	Rigaku ACTOR	Cool Hand Luke	Tecan Evo
Pin	SPINE ALS SSRL	N/A	SPINE ALS SSRL	SPINE ALS SSRL	SPINE ALS SSRL	SPINE ALS SSRL	SPINE ALS SSRL	SPINE ALS SSRL	All pins	SBS trays
Puck	ALS, Unipuck	N/A	ALS, Unipuck, ACTOR	ALS, Unipuck	ALS, Unipuck	ALS, Unipuck	ALS, Unipuck, ACTOR	ALS, Unipuck, ACTOR	Staff assisted	N/A
Sample capacity	24 × 16	20 (fraction collector)	5 × 16	12 × 16	12 × 16	6 × 16	5 × 16	5 × 16	52 pins + ‘infinite’	3 × 384

**Table 2:** SAXS and MALS parameters

	*R*_g_ (Å)	*D*_max_ (Å)	MW_MALS_ (kDa)	MW_SAXS_ (kDa)	MW_theoretical_ (kDa)
Complex	32.4	129	50.8	44.8	41.7
Spytag003-sfGFP	24.8	131	–	34.9	30.4
SpyCatcher003	16.9	57	13.7	13.3	11.3
