# Supplementary material for: ALS-ENABLE: creating synergy and opportunity at the Advanced Light Source synchrotron structural biology beamlines
Source: J Synchrotron Radiat. 2025 Jun 18;32(Pt 4):1059–67. doi: 10.1107/S1600577525004205 (PMC12236258; doi:10.1107/S1600577525004205)
Supplement: Supplementary file 1 [file s-32-01059-sup1.pdf]

JOURNAL OF SYNCHROTRON RADIATION

Volume 32 (2025)

Supporting information for article:

ALS-ENABLE: creating synergy and opportunity at the Advanced Light Source synchrotron structural biology beamlines

Corie Y. Ralston, Sayan Gupta, Joshua T. del Mundo, Aimee Chi Soe, Brandon Russell, Behzad Rad, James Tyler, Sathi Paul, Darren N. Kahan, Line G. Kristensen, Simruthi Subramanian, Savannah Kidd, Kathryn Burnett, Banumathi Sankaran, Scott Classen, Daniil Progozhin, John R. Taylor, Jeff M. Dickert, Kevin B. Royal, Anthony Rozales, Stacey L. Ortega, Marc Allaire, Jay C. Nix, Gregory L. Hura, James M. Holton, Michal Hammel and P. D. Adams

S1. Development of mixing set-up for hybrid spectroscopy-XFMS

Although there are many reports of XFMS studies under equilibrium conditions, the use of time-resolved XFMS is limited. Here we report on the new microfluidic mixing capability developed at ALS beamline 3.3.1 for this study. We developed the mixer with two main considerations. First, the usability of the microfluidic mixer is highly dependent on the repeatable performance of the mixing device, and a major loss of user time can arise from aggregation and clogging in microfluidic mixing channels. Making the microfluidic channel narrower to achieve efficient and ultrafast mixing works well only for well-behaved sample conditions; thus, it is necessary to determine a practical cut-off value when estimating a mixing dead time for robust and repeatable XFMS usage. Here we chose the combination of tubing diameters of 200 μm with the T-mixer U-466S (IDEX), as a compromise between robustness, shorter mixing time and minimum sample usage to monitor the kinetics of SpyCatcher003 binding to SpyTag003. Second, a homogeneously mixed sample is required for XFMS samples. Unlike ultrafast mixing studies with spectroscopy or X-ray scattering experiments where mixed reactions are monitored by focusing the probe directly onto the homogenously mixed portion of the reaction mixture, the hydroxyl radical based mixing requires complete homogenous mixing of the entire sample in the mixing chamber. XFMS or other hydroxyl radical-based approaches collect samples for post-exposure mass spectrometry data analysis. Therefore, prematurely mixed reactants before sample exposure will introduce errors in quantitative LCMS-based data analysis to determine solvent accessibility changes. With these factors to consider, here we report the development of a mixing configuration at ALS beamline 3.3.1 that will work with inline spectroscopy in conjunction with XFMS, and also will provide near single millisecond mixing delays to cover a significant range of bimolecular events without sacrificing reliable and repeatable usage.

S2. Time-resolved mixing configuration

The mixing configuration, as shown in **Figure S1A and B**, has two sample loading pumps that push reagents into the turbulent high flow T-mixer from IDEX Health-Science. The pumps and coordination with X-ray exposure are controlled through a LabVIEW interface. We used two mixing modes: First, continuous flow mixing, where samples are mixed, passed through the X-ray beam, and collected in a fraction collector without any pause. This mode of mixing allowed short mixing delays, ranging from 10 to 400 ms. Second, stopped-flow mixing, where samples are mixed in a mixing loop, paused for a given delay, then passed through the X-ray beam and collected in the fraction collector. This mixing mode gave long delays of 500 ms and higher. In both cases, the sample flow speed was fixed to get the same X-ray dose for all mixing delays. The fluorescence from the mixed samples was collected just prior to X-ray exposure. The total mixing delay is dependent on the flow rate or flow velocity and can be determined from the following equations:

$$t = (\pi r^2 \; l) / Q \quad (1)$$

$$t = l \; / \; v \quad (2)$$

where t is the post-mixing travel time of the solution inside a tube of length l at a flow velocity of v, and a volumetric flow rate of Q. The length of the mixing loop and the position of the XFMS exposure window play important roles in determining the overall delay. Since the spectroscopy probe and X-ray exposure probe window each have a path length along the direction of the flow and are situated successively inline to the flow, the total mixing delay for continuous flow spectroscopy ($t_{spectroscopy}$) is determined by the sum of the travel time up to and including half the length of the spectroscopy probe. Similarly, the total mixing delay for XFMS (t_{XSMS}) is determined by the sum of the travel time up to and including half the length of the XFMS probe. Since the path length for the spectroscopy probe and XFMS probe are small and they are situated very close to each other at a very high flow rate, which is typical for XFMS studies using microfluidics, $t_{spectroscopy}$ is approximately equal to t_{XSMS}. In the current setup, the time difference is 100 μs. The total mixing delay for stopped flow is the sum of t_{XSMS} and the added instrument delay after stopping the flow in the mixing loop.

S3. Establishing mixing efficiency, mixing deadtime, and mixing delays

The optimization aimed to achieve homogeneous mixing at our instrument's shortest length (**l**) between the T-mixer and the X-ray exposure position. In the continuous flow mixing setup, the shortest length is 5 cm, which is currently limited by the stereo mechanical positioning of the T-mixer and the microscope probe and X-ray exposure position on the sample. We mixed yellow and blue pigments to produce green and qualitatively observed the efficiency of achieving a homogenous green color using different mixing flow rates (**Fig. S1C**). The visual observation of the color is done by replacing one of the fluorescence microscopes in the automated sample-handling device with a visible one. We observed that the T-mixer needs at least 3 ml/min of higher flow rate to produce a homogenous green color at the shortest distance of our instrument between the T-mixer and the X-ray exposure position. For a more quantitative view of the mixing efficiency, we mixed 2.5 μM Alexa with 20 mM Tryptophan. Tryptophan is known to scavenge Alexa fluorescence by a non-radiative energy transfer process. Although energy transfer efficiency is low compared to the Tryptophan residue inside a protein, using a relatively higher concentration of Tryptophan relative to the donor Alexa provided a measurable change in the Alexa emission intensity (**Fig. S1E**). Since mixing two of these small molecules in their dilute aqueous solution is a diffusion controlled process, which is at least one hundred times faster than the estimated mixing delay of our instrument, this system was appropriate for testing mixing efficiency vs. flow rate. When equal volumes of Alexa and Tryptophan are mixed at a 2 ml / min flow rate or higher, we observed an immediate decrease in Alexa emission, the same

as that of the value of the premixed solution (**Fig. S1F**). Based on the flow rates, flow diameter, and the distance between the T mixer and the X-ray exposure position, the shortest mixing delay was determined to be around 15 ms with a sample exposure time of 250 μs in the current study. By increasing the flow rate and decreasing the flow diameter, it is possible to reduce the deadtime to 1-2 ms with the current instrument configuration. Furthermore, it is possible to achieve hundreds of microseconds mixing delay by using the high flow speed of liquid jet delivery and reducing the distance between the T-mixer and the XFMS probe.

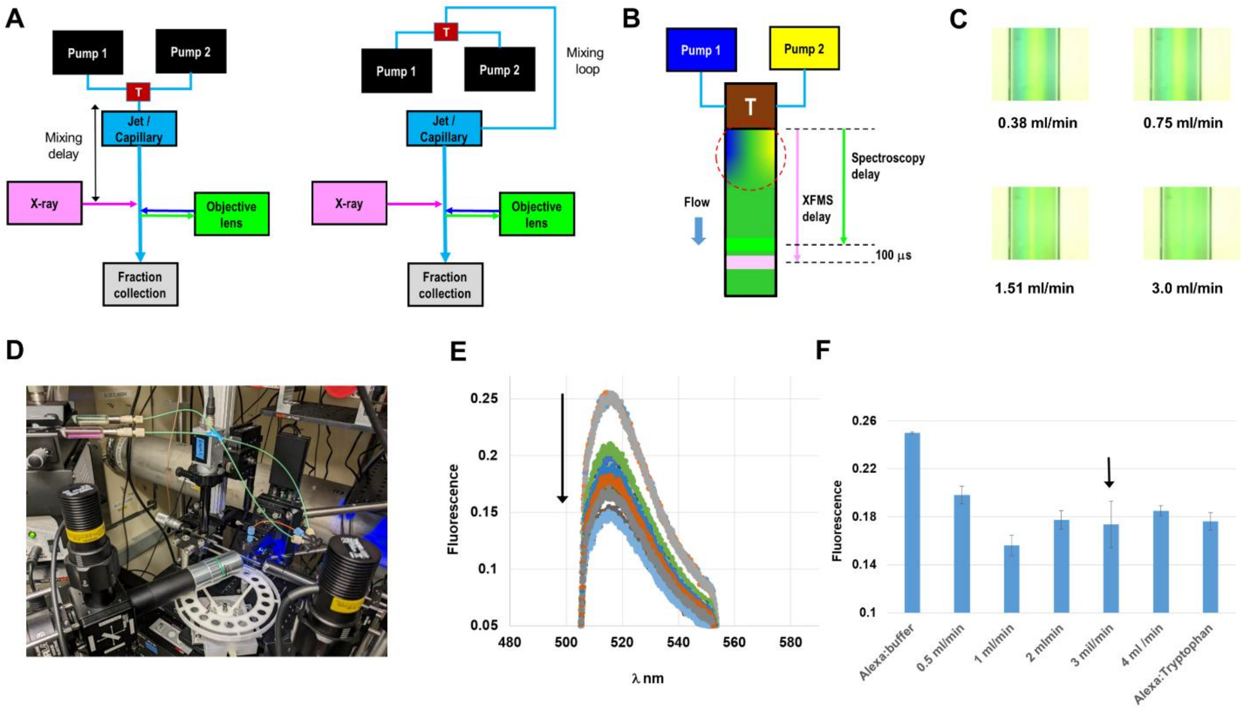

Figure S1 (A) Schematics showing the locations of T mixer (T), sample and mixing loops, jet nozzle or capillary, fluorescence imaging microscope (FIM) and sample exposure window for continuous (left) and stopped flow (right) microfluidic mixing configurations for hybrid XFMS and fluorescence spectroscopy. (B) Close-up schematics of continuous flow mixing cell when used for colorimetric evaluation of mixing efficiency. The dotted region indicates region of mixing inhomogeneity. The position of XFMS and spectroscopy data collection are highlighted to show estimated delay between the hybrid data collection under standard microfluidic configuration which is used for time-resolved hybrid XFMS-spectroscopy data collection. (C) Colorimetric evaluation of optimum flow rate for the maximum mixing efficiency. The 1:1 mixing of blue and yellow dye only provides the homogeneous green color at a flow rate of 3 ml / min. (D) Mixing set-up at beamline 3.3.1 to study kinetics of SpyCatcher003-Alexa555 and SpyTag003-sfGFP binding. (E) The 1:1 (vol./vol.) mixing of 2.5 μM Alexa and 25 mM L-Tryptophan showed near-diffusion controlled quenching of Alexa 488 fluorescence by Tryptophan (downward arrow), which is used to evaluate the optimum flow rate for the maximum mixing efficiency at different mixing flow rates. (F) Loss of Alexa 488 fluorescence at 515 nm when mixed with L-Tryptophan at different mixing flow rates (0.5 to 4 ml /min). The values represented by the bars on the right and left sides of the plot are determined from premixed controls: Alexa 488+ buffer and Alexa 488 + L-Tryptophan.
